# Disturbed resting state EEG synchronization in bipolar disorder: A graph-theoretic analysis^☆^

**DOI:** 10.1016/j.nicl.2013.03.007

**Published:** 2013-03-22

**Authors:** Dae-Jin Kim, Amanda R. Bolbecker, Josselyn Howell, Olga Rass, Olaf Sporns, William P. Hetrick, Alan Breier, Brian F. O'Donnell

**Affiliations:** a Department of Psychological and Brain Sciences, Indiana University, 1101 East 10th Street, Bloomington, IN 47405, USA; b Department of Psychiatry, Indiana University School of Medicine, 340 West 10th Street, Suite 6200, Indianapolis, IN 46202, USA; c Larue D. Carter Memorial Hospital, 2601 Cold Spring Rd, Indianapolis, IN 46222, USA

**Keywords:** *b*, node betweenness centrality, BD, bipolar disorder, *C*, clustering coefficients, DSM-IV, diagnostic and statistical manual of mental disorders, the 4th-edition, DTI, diffusion tensor imaging (image), EEG, electroencephalogram, EOG, electrooculogram, *E_g_*, global efficiency, *E_l_*, local efficiency, FA, fractional anisotropy, FDR, false discovery rate, fMRI, functional magnetic resonance imaging, GABA, gamma-amino butyric acid, *L*, characteristic path length, MADRS, Montgomery–Asberg Depression Rating Scale, MEG, magnetoencephalogram, MRI, magnetic resonance imaging, NBS, network-based statistics, NC, normal healthy control, PLI, phase lag index, *s*, node strength, SCID, Structured Clinical Interview for DSM Disorders, SL, synchronization likelihood, WASI, Wechsler Abbreviated Scale of Intelligence, WM, white matter, YMRS, Young Mania Rating Scale, *γ*, normalized clustering coefficients, *λ*, normalized characteristic path length, *σ*, small-worldness, Bipolar disorder, Electroencephalogram, Synchronization likelihood, Graph theory, Functional connectivity, Resting state

## Abstract

Disruption of functional connectivity may be a key feature of bipolar disorder (BD) which reflects disturbances of synchronization and oscillations within brain networks. We investigated whether the resting electroencephalogram (EEG) in patients with BD showed altered synchronization or network properties. Resting-state EEG was recorded in 57 BD type-I patients and 87 healthy control subjects. Functional connectivity between pairs of EEG channels was measured using synchronization likelihood (SL) for 5 frequency bands (*δ*, *θ*, *α*, *β*, and *γ*). Graph-theoretic analysis was applied to SL over the electrode array to assess network properties. BD patients showed a decrease of mean synchronization in the alpha band, and the decreases were greatest in fronto-central and centro-parietal connections. In addition, the clustering coefficient and global efficiency were decreased in BD patients, whereas the characteristic path length increased. We also found that the normalized characteristic path length and small-worldness were significantly correlated with depression scores in BD patients. These results suggest that BD patients show impaired neural synchronization at rest and a disruption of resting-state functional connectivity.

## Highlights

• Global synchronization of BD patients was reduced in the alpha-band at resting.
• De-synchronized connectivity was localized in fronto-centro-parietal connections.
• Global topology of BD had decreased network clustering and increased path length.
• BD showed the less efficient network processing.
• Network characteristics of BD patients were associated with depression severity.

## 1 Introduction

Bipolar disorder (BD) affects about 1% of the population worldwide and is characterized by extreme variations in mood, motivation, and arousal (Belmaker, 2004). BD is distinguished from unipolar depression by the occurrence of manic or hypomanic symptoms during one or more episodes of the illness. Disturbances of gamma-amino butyric acid (GABA) (Benes and Berretta, 2001; Benes et al., 2000; Petty, 1995; Shiah et al., 1998), monoaminergic (Zubieta et al., 2000) and second messenger systems have been implicated in BD, but the underlying cellular mechanisms remain poorly understood (Belmaker, 2004). Neurobiological markers that are sensitive to the illness might provide a bridge between behavioral and neurobiological abnormalities (Lenox et al., 2002). Accumulating evidence suggests that disturbances in connectivity among brain regions may contribute to brain dysfunction in BD. Deep white matter (WM) hyperintensities are frequently observed in T_2_-weighted magnetic resonance imaging (MRI) (Kempton et al., 2008). Meta-analysis of whole-brain diffusion tensor imaging (DTI) studies demonstrated decreased fractional anisotropy (FA) affecting the right hemisphere WM near the parahippocampal gyrus and cingulate cortex (Vederine et al., 2011). Because signaling among brain regions is dependent on WM tracts, these findings suggest that functional measures of neurotransmission and connectivity would also be affected. Consistent with this prediction, a growing number of imaging studies provide evidences of altered connectivity between brain regions in BD (Calhoun et al., 2011; Frangou, 2011; Houenou et al., 2012). Moreover, there is increasing consensus for decreased connectivity among ventral prefrontal and limbic regions that may reflect a key deficit in BD (Anticevic et al., 2013; Strakowski et al., 2012).

Oscillatory activity may be a general mechanism for the coordination of activity within neural circuits, and disruptions of synchronization among neurons could impact a wide range of cognitive processes (Uhlhaas and Singer, 2010). Disturbed neural synchrony and oscillatory activity may therefore contribute to failures of effective connectivity and neural integration in the illness (Basar and Guntekin, 2008; Uhlhaas and Singer, 2011; Whittington, 2008). While non-invasive measures cannot detect cellular signaling at the level of individual neurons, the electroencephalogram (EEG) and magnetoencephalogram (MEG) can capture the synchronous activity in ensembles of neuronal populations. Moreover, since both EEG and MEG are primarily generated by post-synaptic potentials, they are often sensitive to alterations in neurotransmission secondary to brain dysfunction or pharmacological manipulations (Luck et al., 2011). Thus, these neurophysiological measures have the potential to serve as biomarkers for the disturbances of synchronization and oscillatory activity with high temporal resolution. Waking EEG and MEG activity has received intermittent attention as a potential indicator of altered neurotransmission in BD. A recent review of the BD literature suggests that increased theta and delta and decreased alpha band power are the most robust findings for resting EEG (Degabriele and Lagopoulos, 2009). However, not all studies of EEG power have yielded this pattern of results. For example, greater power in all frequency bands (El-Badri et al., 2001), and increased beta but decreased theta power (Howells et al., 2012) have also been reported.

There are several limitations of previous quantitative EEG studies of BD. One is the reliance on measures of EEG power over a frequency band as the primary unit of analysis. In addition, correlation and coherence among electrode sites are limited to a single time period and may be heavily influenced by volume conduction effects (Nunez et al., 1997). Recently, the measure of synchronization likelihood (SL) has been used to capture the spatio-temporal interactions among electrodes. SL is a novel signal analysis technique that is appropriate for characterizing both linear and nonlinear interdependencies between time series (Stam and van Dijk, 2002) as measured by EEG (Stam et al., 2002, 2003a,b). Hence, it expresses the synchronization of oscillatory activity of each EEG electrode to the other electrodes by measuring the extent to which each pair of electrodes share self-similarity, independent of frequency. When averaged over time, SL produces an index of global synchronization strength with the requisite high temporal resolution to detect rapid fluctuations in synchronization and desynchronization (Montez et al., 2006). Our previous study demonstrated that SL in the resting EEG can detect the fast changing and topologically coherent fluctuations in functional networks in healthy adults (Betzel et al., 2012).

A second limitation in previous EEG studies is the lack of mathematical characterization of the relationship of activity generated across the network of electrodes. Methods derived from the physics of complex systems (Boccaletti et al., 2006; Newman, 2006; Strogatz, 2001) allow structural or functional connections among brain regions to be represented as a comprehensive *network* (Bullmore and Sporns, 2009; Sporns et al., 2005). Graph theory has provided principled mathematical descriptions for the quantitative analysis of complex networks as described by Rubinov and Sporns (2010) and Kaiser (2011). For example, the studies investigating topological organization of the brain has revealed that the human brain network demonstrates *small-world* characteristics at the micro (neuron and synapse) and macro (region and pathway) scales on the brain (Bassett and Bullmore, 2006; Sporns and Zwi, 2004; Watts and Strogatz, 1998) — *i.e.* the network has a greater number of short intra-connections within local sub-networks and relatively fewer inter-connections between sub-networks. A variety of neurological and psychiatric diseases alter connectivity within brain networks (Catani and ffytche, 2005). Graph theory has been utilized to delineate functional and structural network alterations in neurologic disorders such as Alzheimer's disease (He et al., 2008; Stam et al., 2007a, 2009; Supekar et al., 2008), and psychiatric disorders such as schizophrenia (Alexander-Bloch et al., 2012; Fornito et al., 2012; Lynall et al., 2010; van den Heuvel et al., 2010).

To our knowledge, no previous study has investigated the network topologies of resting-state EEG in BD. In this study, we investigated the differences in the organization of functional networks in BD by using a temporal synchronization measure to map the functional connectivity between brain regions and applying graph theoretical analysis to the resting-state SL data. We hypothesized that BD patients would show (1) decreased functional synchronization due to disrupted neural oscillations in the resting-state pathological network; (2) altered network efficiency resulting from the disruption of neuronal coordination; and (3) significant correlations between BD symptom measures and network characteristics.

## 2 Materials and methods

### 2.1 Subjects

Fifty-seven patients with type-I BD (mean age = 41.2 ± 10.5 years, male:female = 25:32) and 87 healthy control subjects (mean age = 40.1 ± 10.6 years, male:female = 35:52) were evaluated. Demographic data are presented in Table 1. All participants were between 20 and 58 years of age and had completed grade-school level education. Patients were recruited through physician referrals from clinics affiliated with the Indiana University School of Medicine in Indianapolis, Indiana, USA. Control participants were recruited using flyers and advertisements. Exclusion criteria for all participants included serious head injury (with loss of consciousness > 5 min), neurological disorders, a history of alcohol or substance dependence in the previous three months, and intoxication (*via* urine screen) at the time of testing. For control participants, exclusion criteria also included a history of substance abuse or dependence, a diagnosis of any current or past Axis I psychiatric illness, or first-degree relatives with BD or schizophrenia. BD patients were diagnosed by doctoral level psychologists (ARB, WPH, BFO) using the Structured Clinical Interview for DSM-IV Axis I Disorders (SCID-I; First et al., 2002), clinical observations, and chart review. Clinical state was assessed using the Montgomery–Asberg Depression Rating Scale (MADRS: Montgomery and Asberg, 1979) and the Young Mania Rating Scale (YMRS: Young et al., 1978). Fifty-three of the 57 patients were receiving psychotropic medication at the time of testing. The types of medication used by BD patients are described in Table 2. The phase of illness was characterized in BD patients by YMRS and MADRS criteria (Berk et al., 2008; Rass et al., 2010). Forty three patients were in a current episode (19 depressed, 10 manic, and 14 mixed) and 14 patients were euthymic. Vocabulary and matrix reasoning subtests of the Wechsler Abbreviated Scale of Intelligence (WASI) were used to estimate intelligence in both groups (Wechsler, 1999). After providing a complete description of the study to all participants, written and verbal informed consents were obtained. The research protocol was approved by the Indiana University–Purdue University Indianapolis Human Subjects Review Committee.

### 2.2 EEG acquisition and preprocessing

EEG signals in the eye-closed resting state were continuously recorded for approximately 120 s using a Neuroscan SYNAMPS recording system (Neuroscan Inc., El Paso, TX) from 29 Ag/AgCl electrodes (Falk-Minow Services, Munich, Germany) with a nose reference based on the International 10–20 system (29 channels = Fp1, Fp2, AFz, Fz, F4, F8, F3, F7, FCz, FC4, FT8, FC3, FT7, Cz, C4, T8, C3, P7, CPz, CP4, CP3, Pz, P4, P8, P3, P7, Oz, O2, and O1). In addition, the vertical and horizontal electro-oculograms (EOGs) were recorded to monitor the eye movements and blinks. Sampling frequency was 1000-Hz with a bandpass of 0.1–200-Hz, and the electrode impedances were less than 10 kΩ. Participants were instructed to keep their eyes closed while resting EEG was recorded for 120-sec. Ocular artifacts recorded by vertical and horizontal EOGs were corrected in the acquired EEG data by the method of Gratton et al. (1983). The EEG was segmented into 2-sec epochs and the epochs with voltage exceeding ± 150 μV were excluded to reduce artifacts. Frequency bands of interest were classified by delta (1–4 Hz), theta (4–8 Hz), alpha (8–12 Hz), beta (12–30 Hz), and gamma (30–50 Hz). Finally, EEG recordings were down-sampled from 1000 to 250 Hz. For each participant, the first 10 artifact-free baseline-corrected epochs (= 5000 samples = 20 s) were selected for further processing.

### 2.3 SL for EEG connectivity

**Fig. 1:**
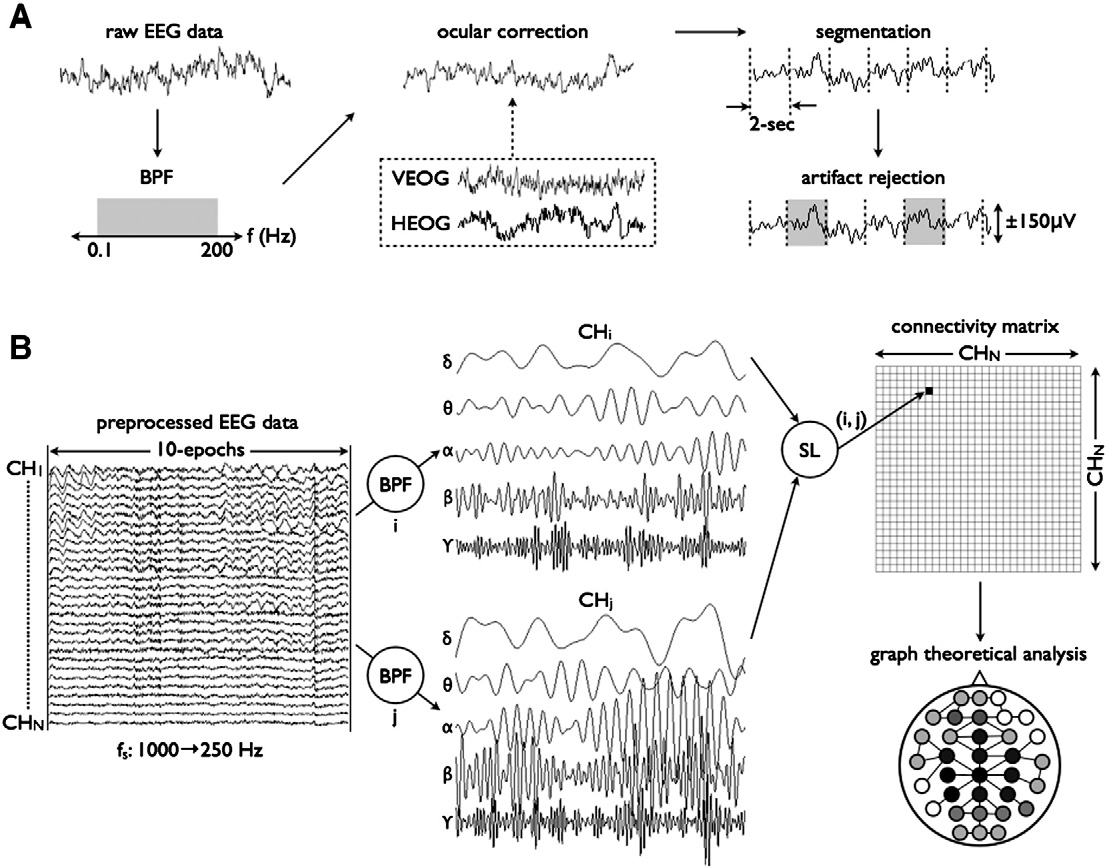
The workflow of all preprocessing for network analysis. (A) The raw eye-closed resting EEG data, first, was band-pass filtered with 0.1 < *f* < 200-Hz, and corrected for the eye movement with references of vertical and horizontal EOG. Then, the EEG was segmented into 2000-ms epochs, where the epochs with voltage samples exceeding ± 150-μV were excluded. (B) Ten epochs (20-sec) were down-sampled from 1000 Hz to 250 Hz, resulting in the time series of 5000 samples for further analysis. The preprocessed EEG data was classified into 5 frequency bands (*δ*, *θ*, *α*, *β*, and *γ*), and the SL was computed between EEG channels resulting in the connectivity matrix for each frequency band. Finally, graph-theoretic analysis was performed, and global/local network measures were calculated.

SL was computed between all pairs of EEG channels in each frequency band as a measure of functional connectivity across time and electrode sites. SL measures generalized synchronization and can detect both the linear and nonlinear inter-dependences between two signals (Stam and van Dijk, 2002). We provide a brief description of its calculation and the strategy for optimal parameter selection in the supplementary material. The result of computing SL for each EEG channel of a specific frequency band is a 29 × 29 matrix where 29 is the number of EEG channels in this study. This weighted matrix provides the basis for computation of network properties among electrode sites. A schematic overview of processing and analysis is shown in Fig. 1.

### 2.4 Network analysis

Graph theoretical analysis was used to quantify network properties (Bullmore and Sporns, 2009). The network consisted of nodes (29 electrodes in this study) and edges (SL value between channels), and was characterized by means of network integration, segregation, and nodal importance. *Network integration* refers to the interactions among specialized brain regions, and represents the ability to combine the information from distributed areas. The path length between brain regions has been used to define the functional integration of the brain (Rubinov and Sporns, 2010). In this study, characteristic path length (*L* and *λ*; the mean shortest path length between all nodes) and global/local efficiency (*E_g_* and *E_l_*) were computed as measures of network integration. *Network segregation* refers to the existence of specialized functional or anatomical regions within a network. The measures of segregation detect the presence of such regions (*i.e.*, cluster) within a network and the presence of network clusters indicates segregated functional dependencies in the brain. We selected the clustering coefficient (*C* and *γ*; the fraction of neighboring nodes being also nodes each other) as a measure of network segregation. The importance of individual nodes was addressed by network centrality. Because the important brain regions usually interact with many other regions, we chose the strength (*s*; the sum of all neighboring connection weights) and betweenness centrality (*b*; the number of shortest paths from all nodes to all others passing through the node) as the centrality measures. Small-worldness (*σ*; to what degree the network is highly clustered with short path lengths) was also computed to characterize the property of integration and segregation simultaneously. Mathematical definitions of the network measures can be found in the supplementary material. To compute the network measures for the 29 × 29 matrix of SL, a fraction of the total number of connections was fixed constant by applying a different threshold for each participant and for each frequency band. The same number of matrix elements was necessary to allow a comparison of network measures obtained from different network topologies (Bullmore and Sporns, 2009). In this study, we constructed the networks from 5% (sparse connection) to 100% (full connection) of maximum connection density.

### 2.5 Correlations between network measures and clinical symptoms

We examined the relationship between the network measures and the clinical state of BD patients statistically controlling for age, gender, and IQ. In this study, the YMRS and MADRS total scores were used as measures of symptom severity.

### 2.6 Statistical analysis

#### 2.6.1 Mean SL

To determine the between-group difference of the extent to which the brain is synchronized at rest, SL values of each participant were averaged across a pair of nodes and the permutation test (Fisher, 1935) was performed as in previous network studies (van den Heuvel et al., 2010; Wang et al., 2010). Briefly, the permutation test is a statistical test for the difference between two means in which the null distribution of test statistic is obtained by a number of random rearrangements (*i.e.* permutations) for each participant's group. First, a *t*-value was calculated for the averaged SL as an observed test statistic between two groups. Then, each participant was randomly reassigned to either patient or control group, resulting in the same number of participants for each group (*i.e.* BD:NC = 57:87 after permutation). The *t*-values were recalculated for the permutated groups 10,000 times, and the null distribution of test statistics was obtained for the group difference. Finally, the proportion of sampled permutations where the *t*-values were greater than the observed test statistic was calculated as the *p*-value of the observed group difference. A level of significance was set *p* < 0.05 (uncorrected), and participant's age, gender, and IQ were used as covariates to remove potential biases to the statistical result.

#### 2.6.2 Connection-wise SL differences

Since the conventional mass-univariate testing might be underpowered with a low contrast-to-noise ratio of the synchronization matrix (Zalesky et al., 2010), network-based statistics (NBS; Zalesky et al., 2010, 2012) were performed to identify the node pairs between which the SL value was significantly changed in BD participants compared to controls. For this purpose, two-sample *t*-tests were independently performed at each synchronization value, and *t*-statistics larger than an uncorrected threshold of *t* = 2.61 (*p* = 0.005) were extracted into a set of supra-threshold connections. Then we identified all connected components in the adjacency matrix of supra-threshold links and saved the number of links. Finally, a permutation test was performed 10,000 times to estimate the null distribution of maximal component size, and the corrected *p*-value was calculated as the proportion of permutations for which the most connected components consists of two or more links. Methodological details for NBS can be found in Zalesky et al. (2010).

#### 2.6.3 Global and local network measures

For global network measures (clustering coefficients *C*, characteristic path length *L*, normalized clustering coefficients *γ*, normalized characteristic path length *λ*, small-worldness *σ*, and global efficiency *E_g_*), permutation tests (10,000 times) were separately performed to determine the between-group differences on each network parameter for the varying thresholds of connection density (5, 10, … , 100%). This procedure followed the same analysis as the permutation tests for the mean SL values (see above, Mean SL). In addition, the role of each node in the SL network was examined by comparing each node-specific local network measures (strength *s*, betweenness centrality *b*, and local efficiency *E_l_*) between BD patients and controls for all EEG nodes.

## 3 Results

### 3.1 Demographics

Demographic and clinical data for the BD patients and healthy control participants are shown in Table 1. Gender, age, and IQ did not differ between groups (all *p* > 0.05), but education differed (*t*_(142)_ = − 3.731, *p* < 0.001) whereby years of education were shorter in BD patients (BD = 12.3 ± 3.2, NC = 14.2 ± 2.9).

### 3.2 SL as a whole connectivity

**Fig. 2:**
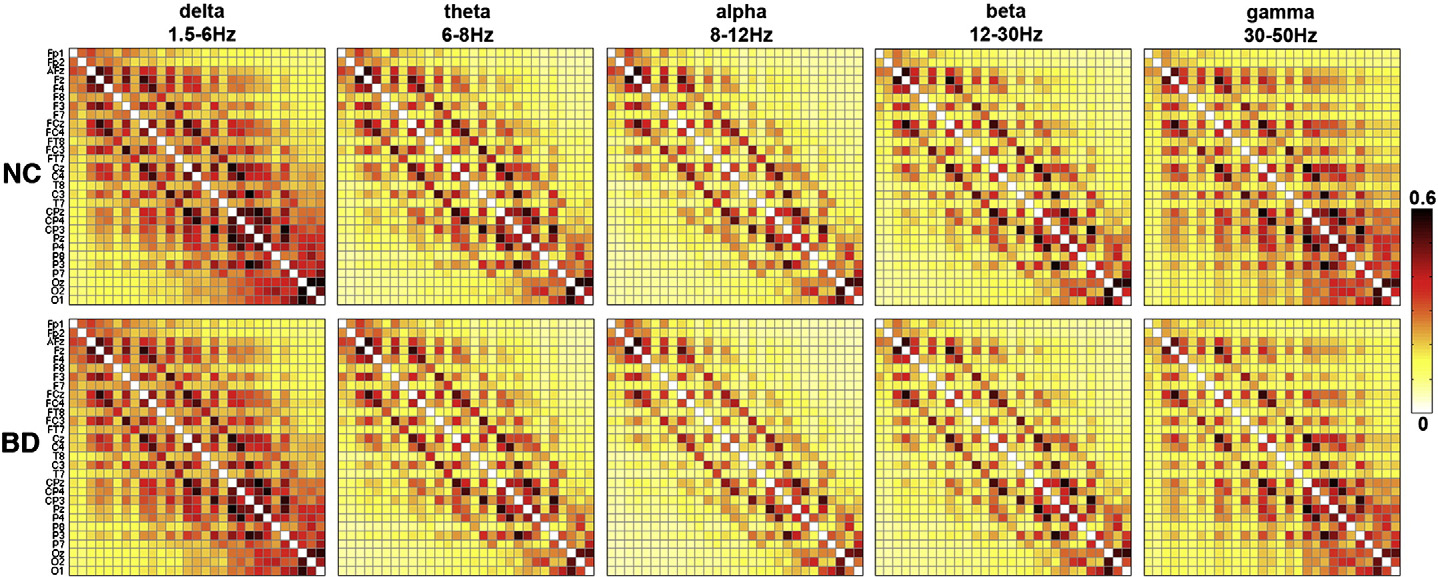
Synchronization matrices across the bipolar disorder (BD) patients (*N* = 57) and the normal healthy control (NC) participants (*N* = 87). The number of EEG channels is 29, resulting in the 29 × 29 square matrix whose elements represent the average strength of SL values across the whole subjects between a pair of EEG channels.

**Fig. 3:**
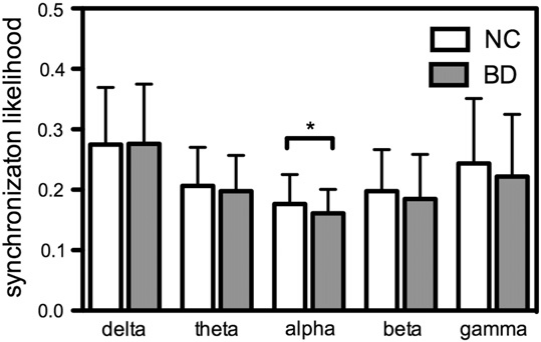
Mean SL of BD patients was decreased (*p* = 0.019, permutation test) in alpha-band as compared to controls.

The SL matrix, using grand-averaged values across BD patients and controls, is shown for each frequency band in Fig. 2. The mean SL in the BD group was decreased (*p* = 0.019, permutation test) in the alpha-band compared to controls (Fig. 3). No between-group differences were found for delta, theta, beta, and gamma frequency bands (all *p* > 0.10).

### 3.3 SL as a local connectivity

**Fig. 4:**
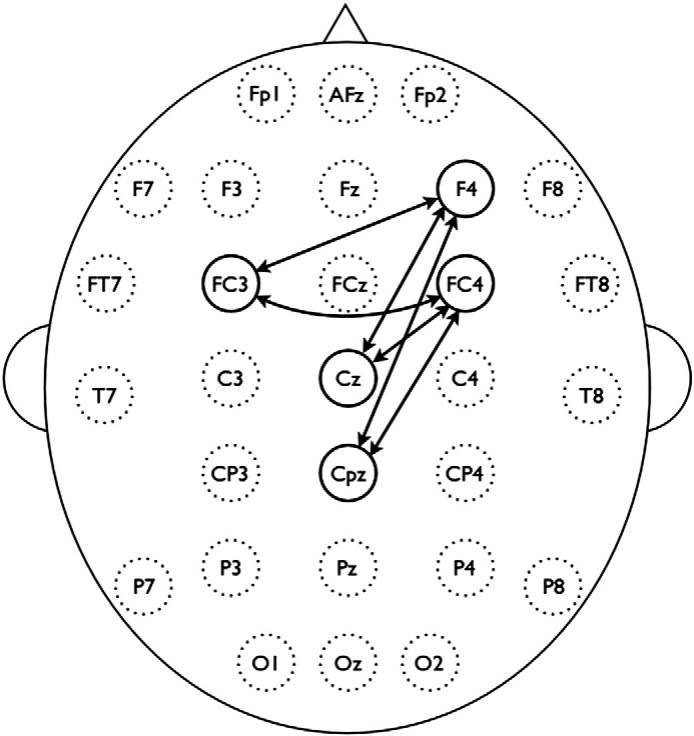
Clustered connections from network-based statistics (NBS). The nodes consisted of F4, FC3, FC4, Cz, and Cpz comprised decreased synchronization in alpha-band of BD subjects compared to controls (*p* < 0.05, corrected).

NBS revealed one localized network (*i.e.* connected and clustered components) in the alpha-band with significantly decreased SL values in BD patients compared to controls (*p* = 0.012, corrected). The nodes consisted of F4, FC3, FC4, Cz, and Cpz, where the inter-hemispheric differences were found in FC3–FC4 and FC3–F4 connections, and the intra-hemispheric differences in Cz–F4, Cz–FC4, Cpz–F4, and Cpz–FC4, respectively (Fig. 4).

### 3.4 Global network properties

**Fig. 5:**
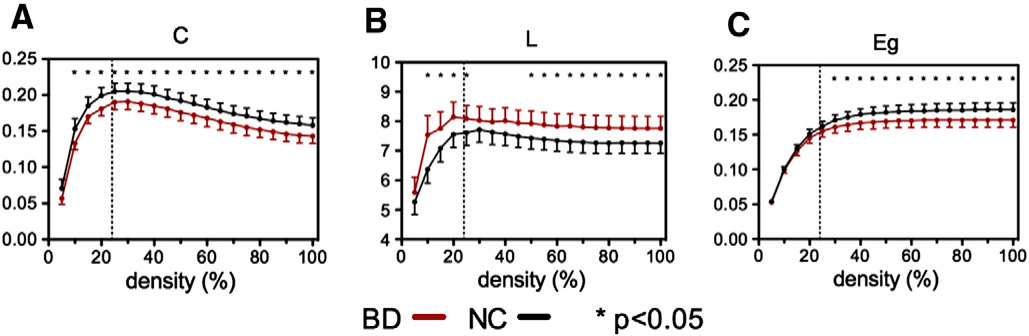
(A) Weighted clustering coefficient *C*, (B) weighted path length *L*, and (C) global efficiency *E_g_* in alpha-band (8–12Hz) for the bipolar disorder patients (BD: red) and healthy controls (NC: black) as a function of connection density. Error bars represent 95% confidence interval, and the asterisks denote where the group difference is significant (*p* < 0.05, permutation test). Vertical dashed line represents the connection density (≈ 24%) from Erdös–Rényi model for 29 nodes, which predicts that most of nodes are fully connected.

In the alpha-band, the networks of BD patients had increased *L* and decreased *C* and *E_g_* as a function of connection density (all *p* < 0.05 with permutation tests). The findings were broadly preserved over a range of thresholds corresponding to connection density of 5–100% (Fig. 5). Because the networks are almost sparsely connected with the highest SL values for a lower threshold of connection density, the corresponding network measures are likely to have the minimum. As the connection density increased, more edges were added into the graphs and the network measures were rapidly increased. Then, beyond the threshold of the Erdös–Rényi model (Erdös and Rényi, 1961), which predicts that most of nodes are fully connected, the network measures tended to converge. SL networks of BD patients showed no significant changes in *γ*, *λ*, and *σ*, which suggest that the networks of BD patients and controls have the same small-world organization for the functional brain network. Also, in other frequency bands, there were no significant changes of global network measures over all thresholds of connection density.

### 3.5 Local network properties

**Fig. 6:**
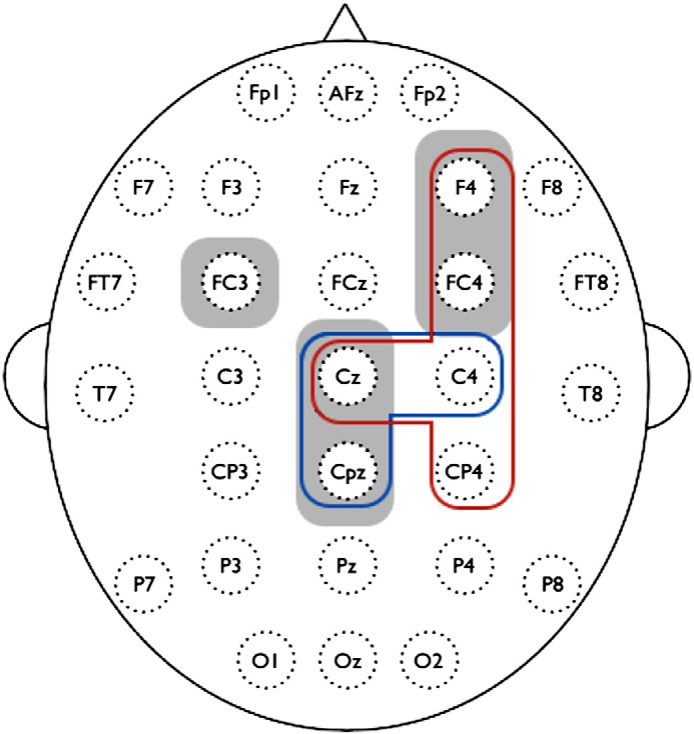
Decreased node-specific network measures in bipolar disorder (BD) patients (*p* < 0.01, uncorrected); red = strength *s*, blue = local efficiency *E_l_*, and gray = nodes of decreased functional subnetwork from the network-based statistics (NBS) in Fig. 4. All nodes but CP4 correspond to alpha-band. Results were computed from the SL matrix at the threshold of 30% connection density, where the clustering coefficient and characteristic path length of the SL network have the maximum values in Fig. 5.

The local network measures for group comparison were extracted from the SL matrix thresholded at 30% of connection density, where the *C* and *λ* of the SL network have the maximum values (Fig. 5). Group comparisons revealed reduced levels of node strength *s* at F4 (BD:NC = 2.96 ± 0.96:3.46 ± 1.32, *p* = 0.005; all *p*-values from permutation test), FC4 (BD:NC = 3.19 ± 1.10:3.75 ± 1.34, *p* = 0.005), Cz (BD:NC = 3.81 ± 1.15:4.36 ± 1.48, *p* = 0.008), C4 (BD:NC = 3.08 ± 0.95:3.56 ± 1.33, *p* = 0.009), and CP4 (BD:NC = 3.77 ± 1.21:4.39 ± 1.73, *p* = 0.01). With the sole exception of electrode site CP4, which showed a difference in the beta band, all other differences were observed only in the alpha band. In addition, local efficiency *E_l_* differed in Cz (BD:NC = 0.27 ± 0.06:0.30 ± 0.08, *p* = 0.007), C4 (BD:NC = 0.26 ± 0.07:0.29 ± 0.08, *p* = 0.009), and CPz (BD:NC = 0.28 ± 0.07:0.31 ± 0.08, *p* = 0.008) in alpha band (Fig. 6). No differences were found for betweenness centrality *b*.

### 3.6 Correlation with clinical variables

**Fig. 7:**
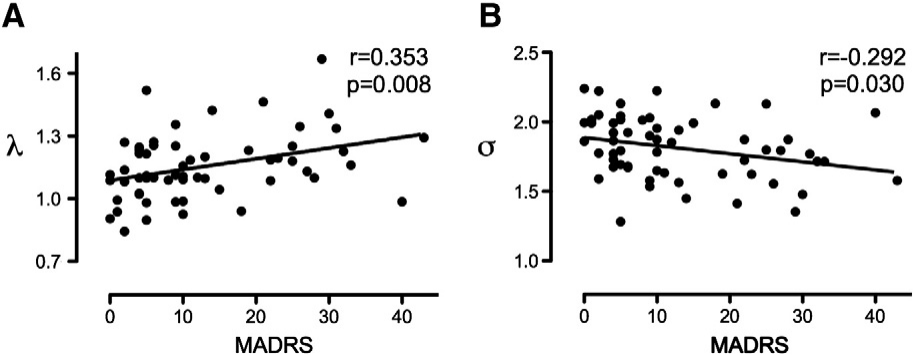
Partial correlations between depressive score of MADRS and (A) normalized characteristic path length (*λ*), and (B) small-worldness (*σ*) in gamma-band for bipolar disorder patients. Correlations for *C*, *L*, *γ*, and *E_g_* were not significant for other frequency bands. Correlations were computed at the threshold of 30% connection density.

To compute the correlations between global network characteristics and clinical symptoms for BD patients, the same threshold was applied to compute network measures for SL matrix with 30% of connection density. Correlation coefficients between MADRS scores and *λ* and *σ* were significant in the gamma band network of BD patients (*λ*: *r* = 0.353, *p* = 0.008, *σ*: *r* = − 0.292, *p* = 0.030) (Fig. 7). However, correlations between MADRS scores and other network measures (*C*, *L*, *γ*, and *E_g_*) were not significant. No significant correlations were found between YMRS and network measures.

## 4 Discussion

This study used SL and graph theoretic analysis to investigate the topological alterations of EEG functional networks in BD patients. Our main findings were: (1) Global synchronization of the resting-state EEG network in BD patients was significantly reduced in the alpha-band; (2) the de-synchronized connectivity in BD patients was localized, predominantly in fronto-central and centro-parietal connections; (3) the global topological organization in BD patients was altered as indicated by decreased network clustering and increased path length, probably resulting in less efficient network processing; and (4) in BD patients, the changes of network characteristics for the gamma band were associated with depression severity. Taken together, our findings support the hypothesis that the functional topological architecture of resting-state brain network is disrupted in BD, especially in the alpha frequency band.

### 4.1 Synchronization in functional brain networks of BD

Our results revealed that the brain networks of BD patients have an altered functional connectivity pattern compared to healthy controls. Specifically, patients showed significantly decreased synchronization for the whole brain network in the alpha-band (Fig. 3). A previous EEG study reported that BD patients showed a generalized pattern of decreased alpha power at rest (Clementz et al., 1994). Whereas the alpha synchronization has been interpreted as idling signals, indicating an absence of information flow among brain areas (Pfurtscheller et al., 1996), recent studies suggest that alpha-wave synchronization reflects active inhibition that is required for efficient performance on cognitive or motor tasks (Herrmann et al., 2004; Sauseng et al., 2005). Because the SL measure in this study indicates how close a node is connected coincidently to other nodes of the network over time, our findings suggest incoherent or desynchronized spontaneous alpha-band activity during resting-state in BD. Furthermore, SL alpha deficits were most prominent in pairwise clusters comprising a subnetwork (Fig. 4) that included the frontal (F4), fronto-central (FC3 and FC4), central (Cz), and centro-parietal (CPz) regions. Decreased functional connectivity was greater over the right hemisphere (F4 and FC4), consistent with a frequent findings of asymmetric alpha band activity in emotional processing and mood disorders (Coan and Allen, 2004). Together, our results demonstrate a breakdown of functional connectivity at rest that likely reflects disrupted information processing and functional integration in BD.

### 4.2 Functional network characteristics of BD

Whereas brain networks often show a preserved small-world property even in neuropsychiatric conditions (Bassett and Bullmore, 2009), some network characteristics are affected. The most typical finding from different diseases and modalities has been an increase in path length, which indicates that patients with brain disorders often have an inefficiently constructed network (Lo et al., 2010; Stam et al., 2007a, 2009; van den Heuvel et al., 2010). Similarly, this study found several altered network parameters (*C*, *L*, and *E_g_*) in BD patients (Fig. 5), despite intact small-world organization. Specifically, the clustering coefficient *C* and global efficiency *E_g_* were significantly decreased in the alpha-band over a wide range of thresholds, while characteristic path length *L* increased. These findings are consistent with a recent tractography-based network study in BD, in which the decreased *C*, increased *L*, and decreased *E_g_* were also reported (Leow et al., 2013). In contrast, recent studies using resting-state fMRI and sleep EEG reported decreased path length and increased network efficiency in major depressive disorder (Leistedt et al., 2009; Zhang et al., 2011). These differences in brain network between mood disorders might be due to a variety of factors, including modality (*e.g.*, EEG, MEG, fMRI, and DTI), selection of network matrix (binary or weighted), number of nodes, applied threshold, different populations, as well as the neurophyisological differences between major depression and BD. In the present study, the changes in topological network properties could be attributable to fragmentation or breakdown of the optimal balance between functional integration and segregation. In addition, these alterations were most apparent at frontal, central, and centro-parietal regions, which highly overlapped with the subnetwork from the NBS (Fig. 6).

### 4.3 Symptom-related effects on functional network organization

Correlations between network metrics and symptom measures in BD subjects revealed that the BD-related alterations of network properties were associated with increased depression. BD patients with longer characteristic path length (*λ*) and diminished small-world characteristics (*σ*) in functional networks had higher depression scores (*i.e.*, MADRS). Given that small-worldness (*σ*) represents the optimal balance between network segregation (*γ*) and integration (*λ*), these results thus indicate a disruption of the functional network integration with increased depressive symptomatology.

### 4.4 Neurophysiological implications

EEG synchronization deficits may index disturbances of GABAergic neurotransmission. Decreased GABA transmission, glutamate receptor expression, and glutamic acid decarboxylase, an enzyme involved in GABA synthesis and regulation, have been implicated in BD (Benes and Berretta, 2001; Beneyto et al., 2007; Brambilla et al., 2003). Several studies have shown compelling evidence of GABAergic modulation of alpha activity. Fingelkurts et al (2004) found that EEG alpha activity was affected by administration of lorazepam, a GABA agonist, in healthy adults. They suggested that GABA signaling can reorganize the neuronal dynamics in alpha band in terms of local and remote functional connectivity. In addition to lorazepam which can reduce alpha activity (Ahveninen et al., 2007), diazepam has also been reported to reduce the eye-closure induced alpha power in MEG recording (Hall et al., 2010). Therefore, the disturbance of EEG synchronization in this study, resulting in the topological alteration of brain network, may reflect dysfunction of GABAergic interneurons in BD patients (Benes and Berretta, 2001). An interpretative issue is the possible effect of medication on GABAergic transmission and EEG activity. Because anticonvulsant mood stabilizers as well as lithium typically upregulate GABA levels or activities, this medication might impact the alpha frequency activity (Benes and Berretta, 2001; Beneyto et al., 2007; Brambilla et al., 2003).

### 4.5 Methodological issues

#### 4.5.1 Interpretation of network alterations

Although the network measures of BD patients were significantly altered in the alpha band (*i.e.*, decreases of *C* and *E_g_*, and increase of *L*), no group differences for *γ* and *λ* were found. Mathematically, because changes in functional connectivity (*i.e.*, decrease of mean SL value) are likely to influence network measures, both *C* and *L* directly depend on the SL weight itself (*cf*. the formula in the supplementary material). Therefore, the lower mean SL of the BD network will result in the decrease of *C* and the longer *L*. However, no significant differences were found for the normalized measures: only non-significant trends for network alterations were observed. It is also possible that BD patients and healthy controls may have their own random counterpart respectively, and that the topological pattern of the BD network (if excluding the weight difference) might not differ from that of healthy controls. BD patients may have subtle topological deficits compared to neurodegenerative diseases that typically show distinct topological differences, such as Alzheimer's disease (Lo et al., 2010; Stam et al., 2007a, 2009).

#### 4.5.2 Network analysis

Although graph theory for network analysis is a promising tool for investigating the topological properties of functional and structural connections within brain, the number of network measures can be influenced by the number of nodes and the average degree in the network (van Wijk et al., 2010). For example, network characteristics computed from different numbers of nodes might differ from each other even if the network topology is not changed. Thus, we chose the same number of nodes (29 channels) for each group to address the node issue. However, the choice of degree is also important to consider in the comparison two networks, even when using a weighted connectivity matrix. Since the raw SL connectivity matrix in this study has continuous values with full connections by mathematical definition, it could also include spurious connections with low SL values. In this case, thresholding and binarization of the network matrix has been a typical approach to eliminate the weak connections. However, without an optimal threshold, the matrix binarization tends to overestimate the contrast-to-noise ratio of the network in that it enhances connectivity values above the threshold and hides the values below the threshold (van Wijk et al., 2010). We therefore chose the weighted network matrix instead of the binary one in the light of several studies showing that results from weighted networks are not different from the binarized matrix (Li et al., 2009; Ponten et al., 2009). Nonetheless, there is currently no gold-standard for selecting the best threshold for the matrix. In the present study, we applied a wide range of threshold values to each SL connectivity matrix, where the number of connections in the SL matrix was fixed according to the connection density (defined as the total number of connections in a matrix divided by the maximum number of connections) from 5 to 100% using increments of 5 (Fig. 5). For lower thresholds, the network tends to split in two or more subnetworks, resulting in the lower *C* and shorter *L*. As the threshold increases, both *C* and *L* also rapidly increase until the network is fully connected. In this study, the full connection was predicted based on the Erdös–Rényi model (Erdös and Rényi, 1961) of random graphs, where a graph with *N* nodes is fully connected if the connection density is larger than 2ln*N*/*N* (≈ 23.2% in this study). For thresholds higher than this value, the network increasingly includes weak and spurious connections, and the value of network metrics tends to saturate or slightly decrease. We found consistent network alterations for global network measures using the varying threshold, while the threshold was fixed with 30% of connection density for local measures and correlations since the networks have the maximum *C* and *L* at that value (Fig. 5).

#### 4.5.3 Network-based statistics (NBS)

NBS is a procedure for non-parametric multiple comparisons to investigate the differences of network connectivity (Zalesky et al., 2010, 2012). This approach is very promising because conventional mass-univariate tests often fail to find connectivity differences because they require adjustment for multiple comparisons (*e.g.* FDR correction). In our initial study (unreported), no significant differences were found from the pairwise comparisons of SL connectivity with 406 connections from 29 nodes and FDR *p* < 0.05, which potentially due to a weak contrast-to-noise ratio in the network (Zalesky et al., 2010). So NBS, a less conservative approach than FDR, was applied which detected a subnetwork consisting of fronto-centro-parietal connections (Fig. 4). It should be noted that no individual connections within this subnetwork were significant with connection-wise comparisons, but only the subnetwork as a whole was significant because NBS are effective only for connected and structured components.

#### 4.5.4 Interpretation of SL

Because the cognitive functions of the brain are fundamentally based on the coordinated interactions of neuronal sources across distributed brain regions, quantifying neural synchrony can provide evidence of functional integration in the brain. Recent neuroscience methodologies have utilized two types of indices for large-scale neural synchrony — linear and nonlinear measures (Pereda et al., 2005). Linear synchrony measures, such as temporal correlation, spectral coherence, and directed transfer function, have been widely used to quantify the degree of synchronization in the neural system. The assumption of the stationarity and linearity between brain activity from different regions limits how well linear measures can characterize pathological nonlinear neuronal synchrony as well as the intrinsic brain dynamics (Aviyente et al., 2011). On the other hand, nonlinear measures address this limitation by using measures of phase synchrony and generalized synchronization. In this study SL was used to detect the linear and nonlinear inter-dependences between two dynamical systems (Stam and van Dijk, 2002). However, some statistical measures for signal interdependencies can be spuriously biased by the volume conduction effect of source activities within the brain (Guevara et al., 2005; Nunez et al., 1997). EEG is sensitive to volume conduction because EEG potentials measured at the scalp are highly dependent on tissue conductivity of activity from one or more sources. In the present study, 29 EEG electrodes were used, which is a relatively small number compared to previous network analyses of MEG data (Buldu et al., 2011; Kitzbichler et al., 2011; Stam et al., 2009). Previous reports suggest that voltage correlations due to volume conduction would be insubstantial for electrodes separated by 4 cm or more (Doesburg et al., 2005; Nunez et al., 1999), and the effect of volume conduction may be reduced with a small number of sparsely-located channels. Further studies are needed to determine an unbiased measure to detect the true neuronal activity between brain nodes or regions. Alternative measures sensitive only to the functional coupling between channels excluding volume conduction have been suggested — *e.g.*, phase lag index (PLI — Stam et al., 2007b; Vinck et al., 2011).

#### 4.5.5 Correspondence of regional alterations

The measured electrical activity on the brain scalp is thought to be primarily generated from common dipole sources within brain. However, source characteristics for EEG activity cannot be identified solely on the basis of scalp recordings. To estimate the orientation and strength of the electrical dipole sources has been known as an ill-posed problem, *i.e.*, there are a number of possible solutions which can explain the measured electrophysiological data. In this study, SL differences from resting EEG data were found in the subnetwork consisting of fronto-centro-parietal scalp regions (Fig. 4). Deeper sulcal sources in right central and precentral sulcal activity of this study could induce a broadly detectable signal despite being a relatively singular generator. So the hypothetical activities might be interpreted as two distinct synchronized cortical nodes rather than one strong deep oscillator. Nunez and colleagues have suggested utilization of surface Laplacian analysis (Nunez et al., 1997, 1999) to investigate radially oriented sources of neural activity in our EEG data and to find the superficial radial dipoles near the brain surface. However, this approach, and other source analysis approaches, have not been validated in conjunction with SL measures, and this goal is beyond the scope of the present clinical study. Importantly, virtually all prior studies using EEG in bipolar disorder, or SL measures in humans, have utilized voltage rather than Laplacian transforms (Boersma et al., 2011; Buldu et al., 2011; Schoonheim et al., 2013; Stam et al., 2007a). It should be noted that the neural synchrony measure by SL has a strong regional correspondence with the local network characteristics (Fig. 6), although these findings cannot unambiguously be related to specific brain sources.

#### 4.5.6 Potential role of education in EEG signal differences

In this study, years of education were lower in the bipolar group (Table 1), although this was not accompanied by a significant difference in estimated IQ between groups. When education level was used as a covariate in the ANCOVA comparing groups, the primary findings of reduced SL and altered network characteristics in the alpha band remained significant. Consequently, it does not appear that variation in education was responsible for SL differences between groups in the present data.

#### 4.5.7 Effects of medications on EEG

Sedative medications could introduce the slowing of EEG frequency (Montagu and Rudolf, 1983). Consequently, anticonvulsants and benzodiazepines in the study could shifts the dominant frequencies of EEG signals in BD patients, and it might have an effect on the comparison of EEG synchronization at rest as well. The possible effects of sedative medications on EEG synchronization cannot be characterized in a cross-sectional study, however. Inclusion of EEG measures in a controlled drug trial could be highly informative in future research.

## 5 Conclusion

This is the first study to investigate the characteristics of BD patients with network-based graph theory using resting-state EEG. In the BD group, there was reduced brain synchronization, resulting in alterations of network topologies, mainly in alpha band, and the connectivity disturbances were found particularly in fronto-central and centro-parietal regions. Network alterations were associated with the depression rating scale of BD patients. Our findings suggest that BD is associated with an abnormal resting state that likely originates from disrupted connectivity, which may be affected by depressive symptom severity.

## Figures and Tables

**Table 1:** Demographic and clinical characteristics of participants.

	Bipolar disorder patients	Healthy control subjects	Statistics	*p*-Value
*N*	57	87		
Gender (male/female)	25/32	35/52	*χ*^2^_(1)_ = 0.187	0.67
Age (years)	41.2 ± 10.5	40.1 ± 10.6	*t*_(142)_ = 0.607	0.54
Education (years)	12.3 ± 3.2	14.2 ± 2.9	*t*_(142)_ = − 3.731	< 0.001
IQ	102.8 ± 17.5	106.1 ± 14.7	*t*_(142)_ = − 1.255	0.21
Age of onset	23.0 ± 7.4			
Duration (years)	18.2 ± 11.8			
YMRS	12.5 ± 10.7			
MADRS	13.0 ± 11.1			

Values represent mean ± standard deviation. Abbreviations: IQ — Intelligence quotient; YMRS — Young Mania Rating Scale; and MADRS — Montgomery-Asberg Depression Scale.

**Table 2:** Medication types of bipolar disorder groups.

Category		Number of patients
Antipsychotic	Atypical	37
	Typical	8
Anticonvulsant		26
Antidepressant		24
Benzodiazepine		16
Lithium		14
Buspirone		3
Stimulant		2
Anticholinergic		4
No medication		4

Note: Patients taking psychotropic medications typically used multiple medications. Antidepressants include SSRIs (n = 8), SNRIs (n = 4), TCAs (n = 2), trazodone (n = 3), mirtazapine (n = 2), and bupropion (n = 4). Four patients were taking 2 types of anticonvulsants, 1 patient was taking 3 types of atypical antipsychotics, 2 patients were taking 2 types of atypical antipsychotics, and 5 patients were taking 2 types of benzodiazepines. The only type of SNRI antidepressant was effexor (venlafaxine).
