# Supplementary material for: Disturbed resting state EEG synchronization in bipolar disorder: A graph-theoretic analysis
Source: Neuroimage Clin. 2013 Mar 22;2:414–23. doi: 10.1016/j.nicl.2013.03.007 (PMC3777715; doi:10.1016/j.nicl.2013.03.007)
Supplement: Supplementary file 1 — Supplementary material. [file mmc1.docx]

**1. Synchronization likelihood (SL)**

SL is a measure of generalized synchronization and can detect the linear and nonlinear inter-dependences between two dynamical systems ([Stam and van Dijk, 2002](#_ENREF_12)). The initial assumption is that the state of a given system, *X*, at a given time, *i*, can be represented using the time-delay embedding technique ([Takens, 1981](#_ENREF_13)):

$$X_{i}=\left\{ x_{i},x_{i+L},x_{i+2\times L},\cdots,x_{i+(m-1)\times L} \right\}$$

where *L* is the time lag and *m* is the embedding dimension. Then, the distance metric (*e.g.* Euclidean distance) at a reference time *i* can be computed with the remaining time *j*:

$$d_{X_{i}\to X_{j}}=\left| X_{i}-X_{j} \right|$$

where the number of state vectors (*Ñ*) should be *N*-(*m*×*L*) for the time series of *N* samples.

The state vectors having a smaller distance than a threshold (*r_X_*) were considered to be the same state with a reference vector - *i.e.*, recurrence. The threshold is chosen so that the percentage (*p_ref_*) of state vectors which are close to the reference vector is constant. Given two dynamical systems of *X* and *Y*, SL between *X* and *Y* at time *i* is the ratio between the number of simultaneous recurrences and that of total recurrences (*n_rec_*) within a time interval:

$${SL}_{i}=\frac{\sum_{j\in W_{i}} H(r_{X}-d_{X_{i}\to X_{j}})H(r_{Y}-d_{Y_{i}\to Y_{j}})}{n_{rec}}$$

where *H* is the Heaviside function: *H*(*X*)=1 if *X*≥0 and *H*(*X*)=0 if *X*<0. The time interval *W_i_* is defined by two windows (*W_1_* and *W_2_*) where the first window *W_1_* is for the Theiler correction ([Theiler, 1986](#_ENREF_14)) and the second *W_2_* for the sufficient number of recurred vectors:

$$w_{i}=\left\{ j | i-\frac{w_{2}}{2}\leq j\leq i-\frac{w_{1}}{2}, i+\frac{w_{1}}{2}\leq j\leq i+\frac{w_{2}}{2} \right\}$$

Since *SL_i_* is a function of time *i*, the final *SL* between *X* and *Y* is usually computed by averaging *SL_i_* over all *i*.

**2. Optimal parameter selection for SL**

It is known that the parameters required for SL calculation depend on the frequency bands of interest ([See details in Montez et al., 2006](#_ENREF_6)). In detail, given the time series having a frequency band of *f_L_<f<f_H_*, the time lag (*L*) should be small enough to capture the fastest oscillations of the time series. In other words, the sampled interval of the state vector should be shorter than that of twice the highest frequency according to the Nyquist sampling theorem. However, we commonly choose the factor of three instead of two with the practical considerations ([Montez et al., 2006](#_ENREF_6); [Smith, 1999](#_ENREF_11)):

$$\frac{f_{s}}{3{\times f}_{H}}\equiv L\leq\frac{f_{s}}{2{\times f}_{H}}$$

where *f_s_* is the sampling frequency.

On the contrary, the embedding dimension (*m*) should be large enough to allow for the perfect reconstruction of the slowest oscillation in time series. But, the factor of two should be changed to one for the same reason mentioned above:

$$\frac{f_{s}}{f_{L}}\equiv(m-1)\times L\geq\frac{f_{s}}{2{\times f}_{L}}$$

The window *W_1_* is required to exclude the adjacent states which are too close to the reference state, in that the autocorrelation effects within time series might underestimate the embedding dimension ([Theiler, 1986](#_ENREF_14)). The size of *W_1_* is usually defined by the two times larger than the length of the embedding vector to recover the slowest oscillation of time series:

$$W_{1}\equiv2\times(m-1)\times L$$

Finally, the window *W_2_* should be large enough to guarantee the number of recurrent states (*n_rec_*) in the time interval within *W_2_* and outside *W_1_* to be the same with the likelihood (*p_ref_*) of two random vectors being close together as follow:

$$\frac{n_{rec}}{W_{2}-W_{1}+1}\equiv p_{ref}$$

In this study, we used the parameters for each frequency band as follow - δ: *L*=41, *m*=13, *W_1_*=904, *W_2_*=1183; θ: *L*=20, *m*=7, *W_1_*=240, *W_2_*=439; α: *L*=13, *m*=6, *W_1_*=130, *W_2_*=329; β: *L*=5, *m*=9, *W_1_*=80, *W_2_*=279; γ: *L*=3, *m*=6, *W_1_*=30, *W_2_*=229. For all frequency bands, *p_ref_* and *n_rec_* were fixed to 0.05 and 10, respectively.

**3. Graph-theoretical measures of network property**

*Strength*. At an individual node of given network, the number of connections (for binary network) or the sum of weights (for weighted network) to that node can represents the importance of node within the network ([Rubinov and Sporns, 2010](#_ENREF_10)). It is defined by $s_{i}=\sum_{j\in N} w_{ij}$where *s_i_* is the strength at node *i*, and *w_ij_* the weight (*i.e.*, *SL* value) between node *i* and *j* of the network *N*, respectively.

*Clustering coefficients.* This metric provides information about the prevalence of clustered connectivity around a given node ([Rubinov and Sporns, 2010](#_ENREF_10)) meaning a degree to which nodes tend to cluster around individual nodes. Weighted clustering coefficient at the *i*-th node (*C_i_*) was defined by the likelihood that the neighbors of a given node are interconnected with each other ([Onnela et al., 2005](#_ENREF_8)) as $C_{i}=\frac{1}{k_{i}(k_{i}-1)}\sum_{j,h\in N} {(w_{ij}w_{ih}w_{jh})}^{1/3}$, where *k_i_* is the degree representing the weighted number of links connected to the *i*-th node. Thus, the weighted clustering coefficient in a given network (*C*) can be defined by the mean of clustering coefficients for the *all* nodes as $C={\sum_{i\in N} C_{i}}/n$, where *n* is the number of nodes in the network.

*Characteristic path length.* A shorter path length between brain regions can represent the stronger potential for structural integration ([Rubinov and Sporns, 2010](#_ENREF_10)). In a given network, the weighted characteristic path length (*L*) is defined by the average of the shortest path length between a given node and the remaining nodes as $L={\sum_{i\in N} \sum_{j\neq i} d_{ij}}/\left( n(n-1) \right)$, where *d_ij_* is the weighted shortest path length between nodes *i* and *j*.

*Small-worldness.* A small-world network represents a network having short node-to-node distance with high clustering ([Watts and Strogatz, 1998](#_ENREF_15)). To compute this measure, the clustering coefficients and characteristic path length should be compared to appropriately constructed random models which typically preserve the local node structure but randomized global topology ([Rubinov and Sporns, 2010](#_ENREF_10)). In this study, we generated 100 degree-matched random networks that preserve the connection weights as well as the number of nodes, edges, and degree sequences of individual networks ([Maslov and Sneppen, 2002](#_ENREF_5)). Then, we normalized the clustering coefficient and characteristic path length by the average of clustering coefficient (*C_r_*) and characteristic path length (*L_r_*) from the population of 100 randomized graphs. Finally, the network small-worldness (how much the network is highly clustered with the short path length) was defined by $\sigma={{(C}/{C_{r})}}/{{(L}/{L_{r}})}=\gamma/\lambda$ ([Humphries and Gurney, 2008](#_ENREF_2)).

*Global and local efficiency*. The efficiency of a network grossly represents the capacity to exchange information ([Latora and Marchiori, 2001](#_ENREF_3)). The global efficiency (*E_g_*) of a whole network is defined by the average shortest path length as $E_{g}=\frac{1}{N(N-1)}\sum_{i\in N} \sum_{j\neq i} \left( d_{ij}^{w} \right)^{-1}$, and local efficiency (*E_l_*) is also defined by $E_{l}=\frac{1}{2}\sum_{i\in N} \frac{1}{k_{i}(k_{i}-1)}\sum_{j,h\in N,j\neq i,} \left( w_{ij}w_{ih}\left( d_{jh}^{w}\left( N_{i} \right) \right)^{-1} \right)^{1/3}$ meaning the average local efficiencies of each node, where $d_{jh}^{w}\left( N_{i} \right)$ is the shortest path length between node *j* and *h* containing neighbors of node *i*. In contrast to the global efficiency, the local efficiency represents the capacity to transfer the information only within the neighbors of a given node, reflecting how much the network is fault tolerant ([Lo et al., 2010](#_ENREF_4)).

*Centrality*. As a measure of how much traffics pass through the node, we computed the betweenness centrality as the fraction of all shortest paths passing through a given node in the network ([Freeman, 1978](#_ENREF_1)): $b(i)=\frac{1}{(n-1)(n-2)}\sum_{\begin{aligned} j,h\in N \\ i\neq j,i\neq h,h\neq j \end{aligned}} {d_{hj}^{i}}/{d_{hj}}$, where $d_{hj}^{i}$ is the shortest path length between node *h* and *j* passing through *i*.

**References**

Freeman LC. (1978): Centrality in social networks: conceptual clarification. Soc. Netw. 1:215-239.

Humphries MD, Gurney K. (2008): Network 'small-world-ness': a quantitative method for determining canonical network equivalence. PLoS One 3(4):e0002051.

Latora V, Marchiori M. (2001): Efficient behavior of small-world networks. Phys Rev Lett 87(19):198701.

Lo CY, Wang PN, Chou KH, Wang J, He Y, Lin CP. (2010): Diffusion tensor tractography reveals abnormal topological organization in structural cortical networks in Alzheimer's disease. J Neurosci 30(50):16876-85.

Maslov S, Sneppen K. (2002): Specificity and stability in topology of protein networks. Science 296(5569):910-3.

Montez T, Linkenkaer-Hansen K, van Dijk BW, Stam CJ. (2006): Synchronization likelihood with explicit time-frequency priors. Neuroimage 33(4):1117-25.

Nunez PL, Silberstein RB, Shi Z, Carpenter MR, Srinivasan R, Tucker DM, Doran SM, Cadusch PJ, Wijesinghe RS. (1999): EEG coherency II: experimental comparisons of multiple measures. Clin Neurophysiol 110(3):469-86.

Onnela JP, Saramaki J, Kertesz J, Kaski K. (2005): Intensity and coherence of motifs in weighted complex networks. Phys Rev E Stat Nonlin Soft Matter Phys 71(6 Pt 2):065103.

Petrov Y. (2012): Anisotropic spherical head model and its application to imaging electric activity of the brain. Phys Rev E Stat Nonlin Soft Matter Phys 86(1 Pt 1):011917.

Rubinov M, Sporns O. (2010): Complex network measures of brain connectivity: uses and interpretations. Neuroimage 52(3):1059-69.

Smith SW. 1999. The scientist and engineer's guide to digital signal processing: California Technical Publishing.

Stam CJ, van Dijk BW. (2002): Synchronization likelihood: an unbiased measure of generalized synchronization in multivariate data sets. Physica D 163:236-251.

Takens F. (1981): Detecting strange attractors in turbulence. Lecture Notes Math 898:366-381.

Theiler J. (1986): Spurious dimension from correlation algorithms applied to limited time-series data. Phys. Rev. A 34:2427-2432.

Watts DJ, Strogatz SH. (1998): Collective dynamics of 'small-world' networks. Nature 393(6684):440-2.
